# Systematic Review of Hospital Readmissions in Stroke Patients

**DOI:** 10.1155/2016/9325368

**Published:** 2016-09-07

**Authors:** Ahsan Rao, Emily Barrow, Sabine Vuik, Ara Darzi, Paul Aylin

**Affiliations:** ^1^School of Public Health, Faculty of Medicine, Dr. Foster Unit, 3 Dorset Rise, London EC4Y 8EN, UK; ^2^Centre for Health Policy, Institute for Global Health Innovation, Imperial College London, QEQM, St Mary's Hospital, Floor 10, Praed Street, London W2 1NY, UK

## Abstract

*Background*. Previous evidence on factors and causes of readmissions associated with high-impact users of stroke is scanty. The aim of the study was to investigate common causes and pattern of short- and long-term readmissions stroke patients by conducting a systematic review of studies using hospital administrative data. Common risk factors associated with the change of readmission rate were also examined.* Methods*. The literature search was conducted from 15 February to 15 March 2016 using various databases, such as Medline, Embase, and Web of Science.* Results*. There were a total of 24 studies (*n* = 2,126,617) included in the review. Only 4 studies assessed causes of readmissions in stroke patients with the follow-up duration from 30 days to 5 years. Common causes of readmissions in majority of the studies were recurrent stroke, infections, and cardiac conditions. Common patient-related risk factors associated with increased readmission rate were age and history of coronary heart disease, heart failure, renal disease, respiratory disease, peripheral arterial disease, and diabetes. Among stroke-related factors, length of stay of index stroke admission was associated with increased readmission rate, followed by bowel incontinence, feeding tube, and urinary catheter.* Conclusion*. Although risk factors and common causes of readmission were identified, none of the previous studies investigated causes and their sequence of readmissions among high-impact stroke users.

## 1. Introduction

Stroke is one of the leading causes of morbidity and mortality in the world and the cost of management of stroke has vastly increased [1]. Most cases occur in people over the age of 65 years [2]. Approximately 80% of stroke cases are ischaemic in nature, while the remainder are haemorrhagic [2]. Ischaemic stroke (IS) is caused by a thromboembolic event in the diseased artery resulting in acute blockage of the blood supply to a region of the brain [2, 3]. Haemorrhagic stroke results from rupture of a blood vessel or an abnormal vascular structure [3]. This results in bleed in the brain. Subarachnoid haemorrhage (SAH) is a subtype of haemorrhagic stroke that is caused by bleeding on the surface of the brain, whereas in intracerebral haemorrhage (ICH) bleeding occurs within the brain tissue [4].

Various aspects of stroke management have evolved in recent years [5]. Regional programs took a range of initiatives to improve outcomes of stroke, including quicker paramedic services to reach specialised stroke units early, rapid scanning, increased use of thrombolysis within 4 hours of stroke onset, and early rehabilitative program [2]. Previous clinical trials and observational studies have not thoroughly analysed the impact of new management strategies on outcomes of stroke due to small patient cohorts and limited follow-up [6].

Hospital administrative data provides an alternative method to evaluate outcomes of stroke [7]. The initial care of stroke is hospital-based and most complications require hospital reviews, admissions, and regular follow-up [6]. This piece of information is recorded in hospital administrative data and can be used to analyse readmissions [7]. Hospital administrative data has been used in recent years to study various aspects of stroke care [8]. It has the advantage of providing a large cohort of patients, which can be followed up for a long period because the data are generally linked by individual identifier [8].

Hospitalisation and readmission are commonly available information in hospital administrative data [9]. The World Health Organisation suggested that the use of imaging, thrombolysis, length of stay in hospital, and duration of rehabilitation vary in different regions giving rise to variation in mortality and readmission rate of stroke patients [10]. The Patient Protection and Affordable Care Act (PPACA) in US penalizes healthcare for increased RR but it is still not clear what proportion of these readmissions are preventable [11]. The patient population has been categorised into two groups, “high-impact” and “low-impact” users, based on the use of healthcare resources [12]. High-impact users form a small subgroup of patients who utilise the majority of healthcare resources with repeated and persistent readmissions to hospital [12]. The evidence on factors and causes of readmissions associated with high-impact users is scanty [9]. Moreover, most of the readmissions are measured within 30 days of a stroke event; hence, it is still to be discovered if the reasons for long-term readmissions are common in different regions [13]. The aim of the study was to review the evidence on common causes and patterns of short- and long-term readmissions in stroke patients by conducting a systematic review of studies using hospital administrative data. Common risk factors associated with changes in readmission rates were also examined.

## 2. Methods

The literature search was conducted from 15 February to 15 March 2016. The following literature databases were used: Embase (1947–2016), Medline (1946–2016), and Web of Science (1950–2016). Various MESH terms were used to identify studies that evaluated outcomes of stroke patients (Table 1). Alternative spellings for the terms were also included in the search, such as “hemorrhage”, “haemorrhagic”, “ischemia”, and “ischemic”. All the terms were combined with option “OR” to include all search titles. All the subheadings were included in the search. Further studies were identified through cross-referencing of studies reviewed initially. Once, various search terms were explored for stroke and readmission, they were combined with “AND” to include relevant studies for review.

The following inclusion criteria were used:

1. Studies looking at an adult patient population over the age of 18 years diagnosed with nontraumatic ischaemic or haemorrhagic stroke.
2. Studies utilising hospital administrative data to assess clinical outcomes following an event of stroke.
3. Studies evaluating readmission of stroke patients due to any cause.

The following exclusion criteria were used:

1. Studies that reported stroke as a complication or adverse event of another disease.
2. Studies that reported cost-effectiveness rather than clinical outcomes of stroke.

**Figure 1:**
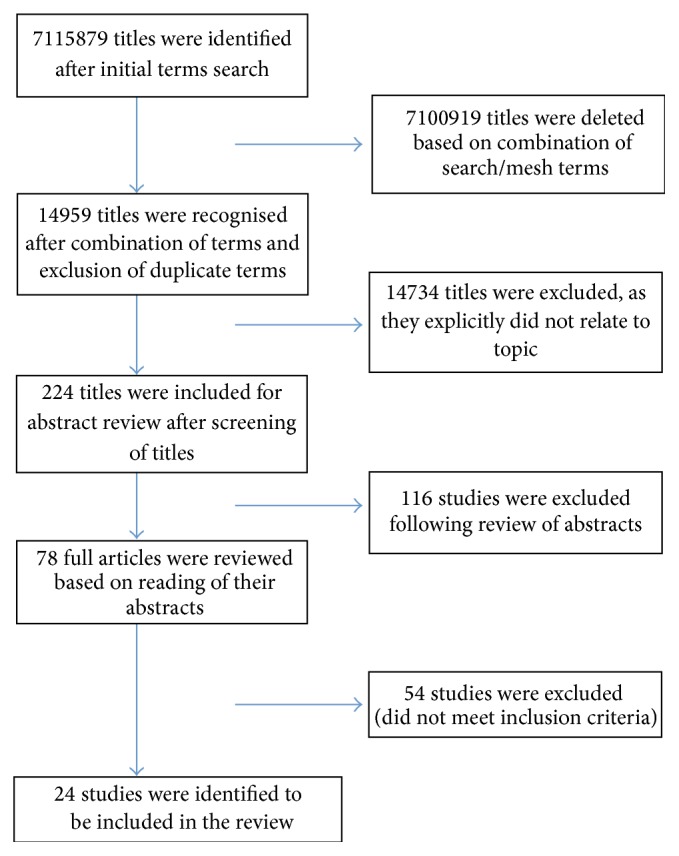
PRISMA diagram for the selection of studies included in the review.

The search strategy for the selection of studies was based on PRISMA protocol, which is primarily used to conduct systematic reviews [14] (Figure 1). Further studies were identified through the cross-referencing of initial studies reviewed. Two independent researchers, EB and AR, reviewed the selected studies separately. Basic demographics were obtained from each study included in the review. Year of study, place of data collection, administrative databases used, and aims and objectives of studies were recorded. Information on methodology of each study was collected, such as number of patients, types of stroke, kinds of readmission rate, and follow-up period. Significant effect of risk factor associated with change in readmission rate was measured as odds ratio (OR), hazards ratio (HR), percentage change, or relative risk (RR), with the confidence interval (CI) of 95%. Ratios and risks adjusted for other confounding factors and having significant impact on RR, *p* < 0.05, were mentioned in the review.

The Newcastle-Ottawa scale was used to assess bias in the studies [15]. The scale uses a star ranking system based on 3 major criteria: selection of participants, comparability, and definition of outcome. A maximum of 8 stars can be obtained by a study and would indicate a low degree of bias. The scale is validated and recommended by Cochrane review methodological guidelines for nonrandomised cohort studies.

## 3. Results

There were a total of 24 studies included in the review (Table 2). Eleven studies selected patients over the age of 65 [9, 13, 16–21], while the rest of the studies included all adult patients. The majority of the studies only included patients with ischaemic stroke. Few studies assessed outcomes in both haemorrhagic and ischaemic stroke patients [4, 6, 18, 21–25]. Eleven studies had high rating on the Newcastle-Ottawa scoring system, 6 and above, suggesting a low risk of bias associated with them [9, 13, 16–19, 22, 23, 25–28]. These studies benefitted from large population-based cohort of stroke patients, selection of a comparison group from the same population, clearly defined outcome measure, comparison of readmission rates between groups, and adequate follow-up. Other studies with low scoring lacked a well-demarcated control group, comparison of outcomes between groups, and complete long-term follow-up.

### 3.1. Causes of Readmission

Four studies evaluated causes of readmissions in stroke patients (Table 3). Common disease-related groups for all-cause 30-day readmission in all types of stroke patients were as follows: kidney and urinary tract infections, simple pneumonia and pleurisy, heart failure and shock, oesophagitis, gastritis, and other gastrointestinal disorders, and, lastly, nutritional and other metabolic disorders [21]. Common causes of 30-day unplanned readmissions in ischaemic stroke patients were recurrent stroke (33%), infections (septicaemia, aspiration pneumonia, pneumonia, and urinary tract infections, collectively 14.5%), and cardiac conditions (dysrhythmia, congestive heart failure, chest pain, and acute myocardial infarction, collectively 10.4%) [11]. Common causes of all-cause 1-year readmissions for haemorrhagic and ischaemic stroke were recurrent stroke (26.3%), infections (15.1%), accidents (9.5%), cardiopulmonary disease (9.1%), cancer (3.5%), diabetes (2.2%), and other causes (32.7%) [6]. In a study with a 5-year follow-up, the causes of readmissions were as follows: pneumonia and respiratory causes (8.6%), acute MI (5%), recurrent stroke (4.9%), gastrointestinal disorders (3.9%), congestive heart failure (3%), other vascular diagnoses (2.9%), cardiothoracic procedures (1.8%), psychiatric disorders (0.4%), hip fractures (0.2%), and other diagnoses (21.8%) [9].

### 3.2. Factors Associated with the Change in the Readmission Rate

Based on the net number of studies mentioning risk factors impacting the readmission rate, common patient-related risk factors associated with increased readmission rate were age and history of coronary heart disease, heart failure, renal disease, respiratory disease, peripheral arterial disease, and diabetes (Table 4). Among stroke-related factors, length of stay of index stroke admission was associated with increased readmission rate, followed by bowel incontinence, feeding tube, and urinary catheter.

Increased age, past medical history of vascular conditions, heart failure, and renal disease were common risk factors associated with the increase in the readmission rate of various types based on studies that reported factors with positive impact on readmission rate (Table 5).

Certain risk factors were found to be associated with reduction in the readmission rate. All-cause 30-day readmission rate was significantly reduced by early decision of not to resuscitate [29], social engagement [29], and higher use of occupational therapy [11]. All-cause 90-day readmission rate was significantly reduced by the use of inpatient rehabilitation [24] and all-cause 1-year readmission rate was declined by the persistent use of antiplatelet therapy [26].

Care of patients in the certified stroke centres [18] and critical access hospitals [19] had no impact on readmission rate. Two studies evaluated 1-year all-cause readmission rate and 1-year readmission for fractures in stroke patients but did not assess risk factors associated with change in readmission rate [30, 31].

## 4. Discussion

This review was based on 24 studies that identified various risk factors and causes of readmission. Prominent causes of readmissions were recurrent stroke, respiratory and urinary infections, acute cardiac conditions, and diabetes. In general, elderly patients with a background of cardiovascular conditions had increased short- and long-term readmission rate. None of the studies evaluated factors and causes of readmission in different subgroups of stroke patients, in particular high-impact users. Studies used different definitions of readmission rate, which made it difficult to compare the data and perform meta-analysis.

The review has focused on studies using administrative data to analyse significant factors and causes of readmission at population level. This may help health policy makers to develop accurate prediction models and management plans at regional level for patients at high risk of readmissions. Previous clinical studies have assessed similar risk factors but they had the disadvantage of limited number of patients and a shorter follow-up period [7, 32]. Calculation of cause-specific readmission rates required a larger sample of patients, possibly with the use of hospital administrative data [25, 33].

The long-term pattern of readmissions in different subgroups of stroke patients could not be deduced from the review. Most of the studies calculated readmission rate with a follow-up of less than one year [13, 16, 27, 28]. Studies with long-term follow-up period merely focused on risk factors associated with higher readmission rate [4, 31]. A minority of studies evaluated common causes of readmission with crude cross-sectional analysis of whole population. No effort was made to categorise patients into subgroups based on rate of readmission and cumulative LOS. This would have been particularly helpful to identify patients with a high-impact use of hospital care or those who have the potential to become high impact [12, 34]. None of the studies investigated the sequence of hospitalisations in stroke patients. Although common causes of readmission were identified, further investigation is needed to find out the timing of occurrence of causes of readmission. If there is a difference in the timing of occurrence of causes of readmission or pattern of hospital care use in subgroups of stroke patients is still uncertain. This could provide vital information as it has been shown that subgroups of pneumonia patients had variable prognosis and time to recovery based on timing of occurrence of complications [35].

The readmission rate was shown to provide relevant and detailed information on the outcome of stroke when the metric was cause-specific, risk-standardised, and assessed unplanned readmissions for preventable causes [6, 9]. Cause-specific readmission rates were mostly calculated for preventable complications, for example, sepsis, recurrent stroke, and fracture [25, 26, 31]. Recurrence rate was a cause-specific readmission rate that measured rate of another stroke event after first stroke. In another study, time to first recurrent stroke was also analysed [26]. The identification of specific causes of readmission based on ICD discharge coding has been shown to be validated and highly sensitive [32].

Measurement of 30-day all-cause readmission to assess the quality of care was recommended by the Affordable Care Act and the Agency of Healthcare Research and Quality (AHRQ) in the US [26]. Hospitals with higher 30-day readmission rates were penalized, with the aim of improving the quality of care by reducing readmission rate. However, a significant proportion of patients after stroke get readmitted for planned procedures, on-going care follow-up, and rehabilitation [11, 29]. The measurement of cause-specific 30-day readmission rate would be better instead, because it can be used to calculate readmission rates due to preventable causes. For example, compliance with antiplatelet therapy was shown to reduce readmission rates for recurrent stroke [29].

Studies have used readmission rate as an outcome measure to assess morbidity in the patients after they had stroke [4, 13, 17–19, 22, 23, 27, 30]. It has been used along with other outcome metrics, such as length of stay, discharge destination, and mortality rate. They have performed separate analysis to evaluate factors impacting the mortality rate of the patients. They mainly focused on assessing annual trends and the impact of various factors on the overall outcome and prognosis of the stroke patients. Hence, factors were recognized which impacted both readmission and mortality rate or just one of them. Since hospitals are penalized for higher readmission rate in some regions, certain studies have only focused on measuring factors associated with higher readmission rate and avoidable causes of readmission [8, 11, 21, 26, 29].

The review had a few limitations. The review only included those studies that used administrative data. The data is collected over a large patient population increasing its generalizability. Most of the studies evaluating causes of readmissions have used administrative data. Other studies using local observational or clinical data had a small patient sample and mainly focused on assessing risk factors associated with the change in the readmission rate. By reviewing studies using administrative data, we have tried to reduce heterogeneity between the studies. However, the selection of stroke patients is based on coding system used by the researchers, which can introduce selection bias and impair its validity. The use of diagnostic coding is prone to errors when used to select cohort of the stroke patients and identifying causes of readmissions. This can lead to bias in the effect measure estimates, such as odds ratio and relative risk. Very few studies evaluated long-term outcomes of stroke. Some studies used the same database; common databases used were Medicare, Medicaid, and Veterans Affairs (VA) administrative data [9, 13, 16, 17, 23, 26, 32]. Readmission rates with multiple definitions were used in the studies, making it difficult for homogenous international comparison. The studies analysing hospital administrative data are prone to selection bias as specific codes are used to identify the cohort of patients from retrospectively collected data and the comparison groups are not randomised [32]. Coding errors have been associated with the use of administrative data [12]. This may affect precise identification of stroke patients and calculation of incidence of complications. In some studies, comparison groups were not matched for confounding factors [4, 9]. Similarly, readmission rates were not risk-standardised to tackle a common problem with the use of administrative data, that is, patient case-mix [26, 33].

Common causes of readmission were identified, but further research is required to find out the sequence and pattern of causes of long-term readmission in stroke patients. Previous studies have evaluated risk factors and causes of readmission for stroke population as a whole [12]. However, it will be more beneficial to investigate risk factors and pattern of readmission in subgroups of patients based on their readmission rate [12].

## Figures and Tables

**Table 1:** MESH terms used for literature search.

1	exp stroke/
2	Brain Ischemia/ or Stroke/ or ischaemic stroke.mp. or Cerebrovascular Disorders/ or Cerebral Infarction/
3	haemorrhagic stroke.mp.
4	Intracranial Hemorrhages/ or hemorrhagic stroke.mp.
5	ischemic stroke.mp.
6	exp hospitalization/
7	hospitalisation.mp.
8	Patient Readmission/ or readmission.mp.
9	readmission rate.mp.
10	Treatment Outcome/ or complication rate.mp.
11	6 or 7 or 8 or 9 or 10
12	1 or 2 or 3 or 4 or 5
*13*	*11 and 12*

**Table 2:** List and characteristics of all studies included in the review.

Study	Year	Place (country)	Population (*n*)	Database source	Type of readmission measure	Bias assessment of study
Sacco et al. [22]	1991	USA	1,034	Statewide Planning and Research Cooperative System (SPARCS)	2-year all-cause readmission	*∗∗∗∗∗∗*
Smith et al. [13]	2006	USA	44,099	Medicare and Medicaid Databases	30-day cause-specific and all-cause readmission	*∗∗∗∗∗∗*
Smith et al. [16]	2005	USA	9003	Medicare	30-day all-cause readmission	*∗∗∗∗∗∗*
Ghose et al. [23]	2005	USA	51,119	Veterans Affair Administrative databases	3-year all-cause readmission and cumulative LOS	*∗∗∗∗∗∗∗∗*
Johansen et al. [30]	2006	Canada	32,107	Canadian Institute for Health Information	1-year all-cause readmission	*∗∗∗∗*
Caro et al. [8]	2006	Canada	18,704	Saskatchewan Health database	5-year all-cause and readmission for cardiovascular	*∗∗∗∗∗*
McGuire et al. [4]	2007	Scotland	9,598	Scottish Medical Record Linkage System	1-yr, 5-yr, and 11-yr readmission	*∗∗∗∗∗*
Bravata et al. [9]	2007	USA	2,603	Medicare and Medicaid Services	5-yr all-cause readmission	*∗∗∗∗∗∗*
Brown et al. [31]	2008	USA	642	Medicaid	Readmission for fractures	*∗∗∗∗∗*
Tseng and Lin [6]	2009	Taiwan	515	Universal National Health Insurance	1-year cause-specific readmission	*∗∗∗∗∗*
Allen et al. [17]	2010	USA	895,916	Medicare	1-year readmission for recurrent stroke	*∗∗∗∗∗∗*
Burke et al. [26]	2010	USA	1413	Medicare and Medicaid Services	1-year readmission (time to recurrent stroke)	*∗∗∗∗∗∗*
Howrey et al. [27]	2011	USA	9185	Medicare	30-day all-cause readmission	*∗∗∗∗∗∗∗*
Gattellari et al. [28]	2011	Australia	26,960	Admitted Patient Data Collection	90-day readmission to calculate cumulative LOS	*∗∗∗∗∗∗*
Lichtman et al. [18]	2011	USA	37,469	Medicare and Medicaid Services	30-day all-cause readmission	*∗∗∗∗∗∗∗*
Lichtman et al. [19]	2012	USA	10,267	Medicare and Medicaid Services	30-day risk-standardised readmission	*∗∗∗∗∗∗∗*
Palmer et al. [32]	2013	England	91,936	HES (Hospital Episode Statistics)	30-day all-cause readmission	*∗∗∗∗*
Lichtman et al. [20]	2013	USA	307,887	Medicare	30-day cause-specific readmission	*∗∗∗∗∗*
Ottenbacher et al. [21]	2014	USA	155,476	Medicare and Medicaid Services	30-day cause-specific readmission	*∗∗∗∗*
Burke et al. [11]	2014	USA	129,676	State Inpatient Database (SID)	Unplanned 30-day readmission	*∗∗∗∗∗*
Chu et al. [33]	2015	USA	17,3966	California State Inpatient Database and State Emergency Department Database	14-day readmission for infective endocarditis	*∗∗∗∗*
Andrews et al. [24]	2015	USA	64,065	State Inpatient database from Arkansas and Florida	90-day all-cause readmission	*∗∗∗∗*
Lewsey et al. [25]	2010	Scotland	51,182	Scottish Morbidity Record Scheme	1-year readmission rate for infective, gastrointestinal, and immobility complications	*∗∗∗∗∗∗*
Fehnel et al. [29]	2015	USA	39,178	Medicare linked to MDS (Minimum Data Set)	30-day all-cause readmission	*∗∗∗∗∗*

**Table 3:** Studies evaluating causes of readmission in stroke patients.

Studies	Primary aim of the study	Type of outcome measure	Type of stroke	Categorisation of causes	Common causes
Bravata et al. 2007 [9]	To examine rates and reasons for 5-year readmission following stroke	5-year all-cause readmissions	Ischaemic stroke	DRG diagnosis	Pneumonia, acute MI, recurrent stroke, gastrointestinal disorders, congestive heart failure, other vascular diagnoses, cardiothoracic procedures, psychiatric disorders, and hip fractures.
Tseng and Lin 2009 [6]	To evaluate population-based readmission rate after stroke	1-year all-cause readmissions	All types (haemorrhagic and ischaemic)	Not mentioned	Recurrent stroke, infections, accidents, cardiopulmonary disease, cancer, and diabetes.
Burke et al. 2014 [11]	To find association between hospital-level practices and readmission rate	30-day unplanned readmissions	Ischaemic stroke	Not mentioned	Recurrent stroke, infections, and cardiac conditions.
Ottenbacher et al. 2014 [21]	To assess readmission rate following postacute inpatient rehabilitation discharge	30-day all-cause readmissions	All types (haemorrhagic and ischaemic)	DRG diagnosis	Urinary tract infection, pneumonia, heart failure and shock, oesophagitis, and gastritis.

**Table 4:** Risk factors and their impact on all-cause, preventable, and cerebrovascular readmission rate.

Risk factors	Number of studies with positive association	Number of studies with negative association	Number of studies with no significant association
*Patient-related factors*			
Age	8	1	
Coronary heart disease	4		
Female gender	4	3	
Heart failure	4	1	
Renal disease	4		
Diabetes	3	1	
Peripheral arterial disease	3		
Respiratory disease	3		
Atrial fibrillation	2	1	
Cancer	2	1	
Comorbidity score	2		
Depression	2	1	
Prior hospitalisations	2		
Socioeconomic status	2		
Alcohol misuse	1		
Anaemia	1		
C difficile infection	1		
Dementia	1	2	
Hypertension	1	3	
Hypotension	1		
Immobility	1		
Marital status, married	1		
Non-Caucasian ethnicity	1	1	1
Parkinson's disease	1		
Pressure ulcers	1		
TIA	1		
Valvular heart disease/prosthetic valves	1		
Do not resuscitate order		1	
Hypercholesterolemia			1
Obesity		1	
Previous stroke		1	
Rheumatic disorders		1	
Smoking		1	
*Stroke-related factors*			
Length of stay	3		
Feeding tube	1		
Bowel incontinence	1		
Urinary catheter	1		
Bladder incontinence		1	
Signs and symptoms score		1	
Aphasia			1
Hemiparesis			1
Aspiration pneumonia			1
*Hospital care related factors*			
District hospitals with critical care access	1		1
Treatment by neurologist	1	1	
Discharge to nursing home		1	1
Specialised stroke centres			1
Inpatient rehabilitation facility		1	1
Use of ICU/HDU			2
Endarterectomy			1
Stenting			1
Gastrostomy			1
Intubation			1
Dialysis			1
Occupational therapy		1	
Thrombolysis			2
Urgent scanning			1

**Table 5:** Studies demonstrating risk factors that increased readmission rate. The rate of increase was described as hazard ratio (HR), odds ratio (OR), relative risk (RR), or increase in the percentage.

Studies	Outcome measured	Risk factors
Chu et al. 2015 [33]	14 d readmission for infective endocarditis	Valvular heart disease (OR 1.5; 95% CI, 0.2–10.8)
		Prosthetic heart valve (OR 15.8; 95%CI, 1.9–129.0)
		Urinary tract infection (OR 3.5; 95% CI, 1.0–12.3)

Lichtman et al. 2013 [20]	All-cause 30 d readmission	Female sex (HR 1.14; 95% CI, 1.08–1.21)
		Age (per year increase) (HR 1.02; 95% CI, 1.01–1.02)
		Diabetes (HR 1.43; 95% CI, 1.35–1.51)
		Congestive heart failure (HR 2.29; 95% CI, 2.15–2.43)
		Myocardial infarction (HR 1.54; 95% CI, 1.29–1.85)
		Peripheral vascular disease (HR 1.14; 95% CI, 1.05–1.24)
		Unstable angina (HR 1.49; 95% CI, 1.11–1.98)
		Protein malnutrition (HR 1.43; 95% CI, 1.23–1.67)
		Dementia (HR 1.10; 95% CI, 1.02–1.20)
		Anaemia (HR 1.43; 95% CI, 1.35–1.51)
		Renal failure (HR 2.31; 95% CI, 2.14–2.48)
		Pneumonia (HR 1.28; 95% CI 1.59–1.42)

Burke et al. 2014 [11]	All-cause 30 d readmission	Use of hospice care (OR 5.86; 95% CI, 1.13–30.3)

Howrey et al. 2011 [27]	All-cause 30 d readmission	Hospitalist care (versus nonhospitalist) (HR 1.30; 95% CI, 1.11–1.52)

Smith et al. 2005 [16]	All-cause 30 d readmission	Care by health maintenance organisation (HMO) compared to Fee-For-Service (FFS) health insurance (HR 1.45; 95%, 1.14–1.83)

Smith et al. 2006 [13]	30 d readmission for atherosclerotic diseases	Care by neurologist (versus generalist) (HR 1.17; 95% CI, 1.02–1.34)

Fehnel et al. 2015 [29]	Unplanned 30-day readmission	Bowel incontinence (OR 1.16; 95% CI, 1.06–1.28)
		Feeding tube (OR 1.21; 95% CI, 1.08–1.35)
		Chronic obstruction pulmonary disease (OR 1.26; 95% CI, 1.16–1.38)
		Renal disease (OR 1.26; 95% CI, 1.13–1.42)
		Heart failure (OR 1.17; 95% CI, 1.08–1.27)
		Pressure ulcer grade 4 (OR 1.33; 95% CI, 1.09–1.62)

Tseng and Lin 2009 [6]	1 yr all-cause readmission	Care by nonneurologist (versus neurologist) (OR 1.81; 95% CI, 1.13–2.88)

Allen et al. 2010 [17]	1 yr readmission for recurrent stroke	Prior hospital admissions (RR 12.64; 95% CI, 9.24–17.29)
		High comorbidity score (RR 2.28; 95% CI, 1.18–2.90)

Lewsey et al. 2010 [25]	1-year readmissions for infections, gastrointestinal, and immobility complications	Heart failure (HR 1.19; 95% CI, 110–1.30)
		Renal failure (HR 1.23; 95% CI, 1.10–1.38)
		Respiratory disease (HR 1.24; 95% CI, 1.15–1.33)
		Increased age (HR 2.08; 95% CI, 1.87–2.31)
		Higher socioeconomic deprivation (HR 1.16; 95% CI, 1.08–1.26)
		Prior complications of infections (HR 1.31; 95% CI, 1.24–1.38)
		Prior gastrointestinal complications (HR 1.58; 95% CI, 1.51–1.66)
		Prior immobility complications (HR 1.23; 95% CI, 1.16–1.30)

Ghose et al. 2005 [23]	3 yr all-cause readmission rate	Depression during stroke (log-OR 2.09; 95% CI, 2.03–2.16)

Caro et al. 2006 [8]	5 yr all-cause readmission rate	↑36.2% (95% CI, 30.3%–42.4%) for age (>65 years)
		↑18.1% (95% CI, 14.1%–22.2%) for male sex
		↑20.9% (95% CI, 13.9%–28.4%) for atrial fibrillation
		↑21.6% (95% CI, 16.6%–26.8%) for heart failure
		↑12.1% (95% CI, 7.6%–17.8%) for angina
		↑10.1% (95% CI, 2.7%–14.8%) for prior myocardial infarction
		↑14.0% (95% CI, 9.7%–18.7%) for hypertension
		↑4% (95% CI, 0.2%–8.1%) for transient ischaemic attack

## Abbreviations

RR: Relative risk
OR: Odds ratio
HR: Hazards ratio
NOS: Newcastle-Ottawa scale
CI: Confidence interval
LOS: Length of stay
cLOS: Cumulative length of stay
CHF: Congestive heart failure
MI: Myocardial infarction
RF: Renal failure
AF: Atrial fibrillation
COPD: Chronic obstructive pulmonary disease
TIA: Transient ischaemic attack
SAH: Subarachnoid haemorrhage
ICH: Intracerebral haemorrhage
CAH: Critical access hospital
AHRQ: Agency of Healthcare Research and Quality
ICD: International classification of diseases
VA: Veterans affairs.

## Additional Points

*Availability of Data and Materials.* The study was review of previously published studies.
